# Small bowel obstruction after gastric by-pass: Diagnostic limits and percutaneous drain. A paradigmatic case

**DOI:** 10.1016/j.ijscr.2019.02.036

**Published:** 2019-03-05

**Authors:** Vincenzo Pilone, Mafalda Romano, Michele Renzulli, Carmen Cutolo, Salvatore Tramontano

**Affiliations:** General and Emergency Surgery Unit of Fucito Hospital, University Hospital of Salerno, Italy

**Keywords:** GBP, gastric bypass, SBO, small bowel obstruction, BMI, body mass index, %EWL, percent weight loss, CT, computed tomography, IH, internal hernia, Bowel obstruction, Bariatric surgery, Gastric by-pass, Internal hernia, Obesity

## Abstract

**Background:**

Obesity is showing a constant increase worldwide, making it the major public health problem. Bariatric surgery seems to be the best treatment for these to achieve a good excess weight loss. Gastric by-pass (GBP) is one the most important bariatric procedure, but there are a lot of complications as bowel obstruction for internal hernia, fistula and intussusception.

**Case report:**

We present a peculiar case report of a 53-years old woman that underwent to GBP. On the fourth postoperative day, she presented a bowel obstruction and underwent a second surgical intervention for incisional hernia. Another surgical revision was necessary because of dilatation of excluded stomach. Subsequent external drain of excluded stomach solved the paralytic ileum post bowel obstruction.

**Conclusion:**

Postoperative occlusion is more complex in bariatric patients, either for variety of etiology, either for choosing best approach to the new anatomy. In addition, post-surgery course may be different, because GBP may determine many modifications on gastrointestinal motility and clinical symptoms. An accurate knowledge of bariatric surgery is mandatory in these situations, obtaining the best management.

## Highlights

• We present a peculiar case report of a 53-years old woman that presented bowel obstruction after gastric bypass (GBP), initially for incisional hernia, but another surgical revision was necessary because of internal hernia with dilatation of excluded stomach. A subsequent external drain of excluded stomach solved paralytic ileum post bowel obstruction.
• Post-GBP small bowel obstruction presented many diagnostic limits, in relation to type of surgery and of patient, which often has not typical clinical features.
• Our report underline risk of postoperative bowel distension, that, associated to paralytic ileum, determined an high-risk condition, with hepatic failure. Percutaneous drain only ameliorated condition and, finally, led to resolution.
• Surgical revision should be performed after a comprehensive prediction of possible complications, and postoperative course may present unexpected evolution, as hepatic failure related to lack of peristalsis.

## 1 Introduction

**Fig. 1:**
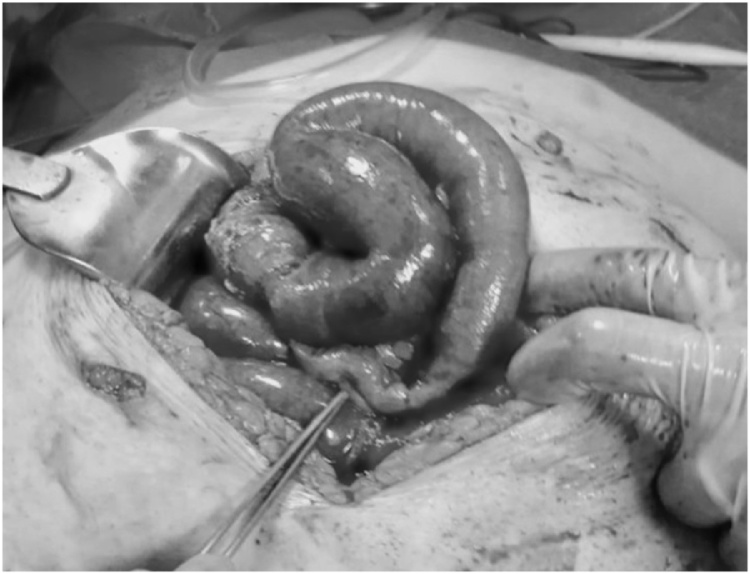
Intraoperative findings of small bowel obstruction after GBP.

Laparoscopic Roux-in Y gastric bypass (GBP) is currently one of the most diffuse surgical options to treat morbid obesity [1]. The occlusive post-GBP complications are mainly related with adherence, internal hernias or intussusception [2]. Small bowel obstruction (SBO) after GBP occurs with a frequency of 0.2–4.5%, years or months after surgery [3]. Among the SBO, internal hernias [2] represent the most common cause: the unique anatomical changes with GBP creates a number of mesenteric defects and possible retrograde peristalsis [1,3]. We presented a paradigmatic case of an early post-GBP occlusion, a life threatening condition, related with late recovery of peristalsis, solved with percutaneous drain of excluded stomach. This work has been reported in line with the SCARE criteria [4] (Fig. 1).

## 2 Presentation of case

A 53-year-old female subject with a BMI 46.6 kg/m^2^ (weight 127 kg, height 165 cm) and gynoid obesity underwent GBP at our university hospital. Patient was suffering with diabetes and hypertension; she had also previously undergone several abdominal interventions: laparoscopic cholecystectomy, umbilical hernia repair and two cesarean sections. After a preoperative multidisciplinary evaluation, the patient was scheduled for a gastric bypass due to the presence of hiatal hernia with gastro-esophageal reflux symptoms. During the operation, a large para-umbilical hernia was found. The omental content was reduced into the abdomen and the conventional gastric bypass procedure was completed. On the second post-operative day, the patient complained with nausea and vomiting. Clinical examination was normal, the abdomen was soft, blood pressure and heart rate were normal. There were no signs of obstruction and bowel was open to air and stool. On post-operative day four, patient was submitted to an x-ray with oral contrast and then to a CT of the abdomen. Radiologist detected an umbilical hernia with engagement of two loops of small intestine surrounded by fluid. While regular progression of the contrast to biliodigestive anastomosis was maintained. It was decided to perform an open surgical exploration. A voluminous para- umbilical hernia was found (in the same place where it was reduced in the previous operation). Color of the incarcerated loop was normal (photo1); it was reduced in the abdomen and an extensive repair the wall defect with interrupted stitches. After a few hours, bowel were again open to gas and stool and clinical condition improved shortly after shortly after surgery. The day after the reoperation a liquid diet was started and on the 4th day after surgery another CT with oral contrast was performed, which discovered regular gastrointestinal transit Discharge was scheduled on postoperative day 7 and an outpatient appointment was booked. After 3 days from discharge, patient returned to the accident and emergency unit complaining with back pain and general discomfort. The patient underwent blood tests and CT of the abdomen. Blood tests showed an alteration of the hepatic profile with an increase in cholestasis indexes The abdomen CT showed "marked distension of bilio-pancreatic loop and of the excluded stomach. adherent bridle and initial signs of suffering from stasis of the loops upstream”. It was decided to send the patient to another surgical exploration. An internal hernia (IH) was found in the Petersen space with free fluid in the abdomen and distension of the excluded stomach and of the small bowel. IH was reduced and the entero - enteric anastomosis was de-rotated and refashioned. Enterotomy on the biliary loop and alimentary loop for decompression, was performed. Extensive adesiolysis was performed and three drainages were left (one in Douglas space, one under mesocolic space, one perianastomotic). The patient was admitted to intensive care unit, due to the unstable general clinical conditions. The patient presented a right pulmonary thickening and hepatic failure. Despite the initial general improvement, cholestasis index and total bilirubin continued to increase (Table 1). At the CT scan, persistent distension of the excluded stomach and of bilio-digestive distension. On the fifth post-operative day, we decided to place an external, eco-guided gastric drain. About 1 liter of bile content was released from the drain. In the following days the patient has a noticeable improvement, with a reduction in cholestasis and biliary indices (Table 1). Another CT abdomen showed "clear reduction of distension of the stomach and of the small bowel”. The patient was discharged on the 12th post-operative day in good condition. Weight at discharge was 122 kg. At the first outpatient appointment, one month after second discharge, the patient had a weight of 115 kg.

**Table 1:** Laboratoristic profile after percutaneous drain (see text).

	Total Bilirubine (mg/dl)	PLA (mg/dl)	ƔGT (mg/dl)
1 POD	1.92	232	180
2 POD	2.56	275	230
3 POD	5.97	349	254
4 POD	12.86	792	300
DRAINAGE	22.43	1218	315
6 POD	8.55	639	152
7 POD	6.35	600	130
8 POD	4.42	485	90
9 POD	3.01	400	87
10 POD	2.92	385	80
11 POD	3.26	300	76
12 POD	3.18	262	60
13 POD	2.74	202	55
14 POD	1.79	175	48

POD: post-operative day.

ƔGT: Gamme-glutamil transferase.

PLA: Alkaline phosphatase.

## 3 Discussion

**Fig. 2:**
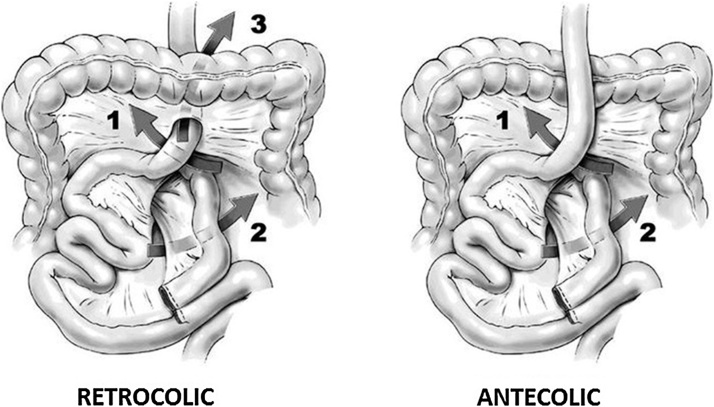
Arrows 1–3 indicate potential spaces for hernia formation: (1) Petersen's space, between the Roux limb mesentery and the transverse mesocolon; (2) the mesenteric opening at the biliopancreatic limb; and (3) the opening through the transverse mesentery when the bypass is in retrocolic fashion. (*Image from Kim Y, Crookes PF. Complications of bariatric surgery. In: Essentials and Controversies in Bariatric Surgery. Huang, C-K, ed. © 2014 Kim Y, Crookes PF. Published under CC BY 3.0 license. Available from:*https://doi.org/10.5772/58920).

We report this complicated case for evidencing, primarily, diagnostic limits of postoperative obstruction, mostly for bariatric case. Secondarily, we underline effect of postoperative bowel distension, that, associated to paralytic ileum, determined a high-risk condition, with hepatic failure. Percutaneous drain only ameliorated condition and, finally, led to resolution. In fact, as shown in the graph, the indexes of cholestasis and bilirubin had decreased in the days following the drainage. Bowel obstruction due to IH, reported during second surgical revision, is a well-known complication [1,2] of laparoscopic Roux-en-Y GBP where usually parts of the bowel pass through a mesenteric defect [3]. In reported case series incidence varies from 0% to about 9% [5,6]. These spaces can develop after GBP due to the rearrangement of the bowel leading to incarceration [5] or strangulation of a bowel loop. IH after GBP arise at typical locations, which are displayed in Fig. 2. These spaces may lead to incarceration or strangulation of the herniated bowel loop. Reported incidence of IH after GBP ranges widely, from 0.2% to 9% [7]. The incidence also depends on the surgical method, with the retrocolic route of the alimentary-limb causing more IH than the antecolic route [8]. In antecolic antegastric GBP, there are two primary locations of IH [9]: an open space between the alimentary limb and transverse colon (called Petersen's space) or a mesojejunal space at the jejunojejunostomy. In the less commonly performed retrocolic configuration of the gastroenterostomy, there is a third space, behind the transverse colon, that can lead to a mesocolic hernia [10]. The retrocolic approach, where a third hernia site is possible (mesocolic hernia), is associated with a higher risk for IH [9]. Differences in surgical techniques also influence the incidence and distribution of hernia. Indeed, the aim of an antecolic approach is to reduce the rate of internal herniation. IH after GBP typically occurs after significant weight loss. Loss of mesenteric fat leads to a widening of intermesenteric spaces. Rapid excess weight loss seems to increase the risk for hernia occurrence [11]. Another situation with changing intra-abdominal anatomy is pregnancy. There are several case reports and series describing IH in patients after GBP with also fatal outcome for the mother and fetus [12,13]. The clinical presentation of IH ranges from intermittent pain, often in the left upper abdomen through more constant abdominal pain, with or without nausea and vomiting to severe, acute abdominal pain [14]. A certain diagnosis can most often only be made by laparoscopy. Symptoms of IH are unspecific. If bowel incarceration is transient, common presenting symptoms are chronic intermittent pain. In the case of acute incarceration, patients may present with symptoms of acute SBO [15]. Particularly the last-mentioned scenario often leads to emergency admissions where fast and efficient diagnostic workup is crucial to reduce morbidity and mortality associated with bowel necrosis and sepsis. Laboratory findings do usually not help in the diagnostic workup, as they mostly reveal no abnormalities. Radiological imaging may comprise plain abdominal X-rays, upper gastrointestinal series, ultrasounds and abdominal CTs. The CT scan is the gold standard for IH but can also be non-diagnostic, especially in asymptomatic patients [16]. In the majority of cases there are no direct signs of but typical indirect signs like a mesenteric swirl sing. A CT scan may also help to distinguish between the different hernia types. It is important to know that CT scan may also be normal even in symptomatic patients.

There is an ongoing debate among bariatric surgeons if primary closure of mesenteric defects reduces the risk for IH. Interestingly, there are studies that showed very low incidence of IH after GBP with no primary closure and therefore, the authors advise against routine closure of mesenteric defects [9,10]. However, there is increasing evidence, that primary closure can reduce the incidence of IH. De la Cruz-Muñoz [7] and colleagues reviewed 2079 patients after GBP and compared IH incidence before and after they started to routinely close mesenteric defects at the initial surgery. They found a significant reduction of IH incidence [17]. Closing mesenteric defects may also bear a risk for complications; however, we believe the benefit outbalances the risk. Primary closure of the mesenteric defects seems to reduce the incidence of IH [18] but can also be associated with complications. Suture of the mesentery, haematomas can occur, causing circulation impairment to the intestine. Closure of the mesenteric defects can also result in increased rate of obstruction at the entero-anastomosis caused by adhesion or rotation of the anastomosis [18]. While it is recommended to use non-absorbable sutures for closing the defects, it is not clear if running of interrupted sutures are superior. It is suggested that pregnancy [19] can predispose individuals to IH formation because of anatomic changes. The enlargement of the uterus and increased abdominal pressure combined with weight loss due to GBP may lead to displacement of the small intestine and internal herniation. Several cases of internal herniation during pregnancy have been reported, and maternal and fetal deaths have been described [8,11].

In our case, we did not close mesentery defects and we had an IH, but, after second reoperation, bowel obstruction was determinate by the failure of recovery of peristaltis, caused by the abnormal expansion of bilio-pancreatic limb and of excluding stomach. This paralitic ileum was a consequence of the incisional paraumbelical hernia, treated in the second surgical intervention. The resolution of bowel obstruction has been realized thorough the drainage of excluded stomach.

## 4 Conclusion

Postoperative occlusion is more complex in bariatric patients, either for variety of etiology, either for choosing best approach to the new anatomy. Drainage of the excluded stomach during SBOs after GBP may solve the paralytic post-occlusion intestinal ileum. An accurate knowledge of bariatric surgery is mandatory in these situations, obtaining the best management.

## Conflicts of interest

No conflict of interest for all authors.

## Sources of funding

No funding was requested.

## Ethical approval

No specific ethic approval was necessary, because case report is related to an operative procedure necessary for health of patient. Moreover, a specific informed consent and authorization for publication of anonymous data was obtained from patient.

## Consent

Specific consent was obtained and specified into the text, with anonymous data. A specific statement is inserted at the end of manuscript. All data related to patient are anonymous.

## Author contribution

Each author contributed to diagnosis, treatment and postoperative follow-up of patient. Specific recording of pathologic data and an adequate review of literature was performed by each author.

## Registration of research studies

N/A.

## Guarantor

A guarantor of data is corresponding author, Dr. Salvatore Tramontano.

## Provenance and peer review

Not commissioned, externally peer reviewed.
